# X-linked congenital adrenal hypoplasia: a case presentation

**DOI:** 10.1186/s12902-021-00785-8

**Published:** 2021-06-15

**Authors:** Hong Ouyang, Bo Chen, Na Wu, Ling Li, Runyu Du, Meichen Qian, Wenshu Yu, Yujing He, Xinyan Liu

**Affiliations:** 1 Department of Endocrinology, Shengjing Hospital of China Medical University, Shenyang, China; 2 Department of Endocrinology, The First People’s Hospital of Kerqin District, Tongliao, Inner Mongolia China; 3 Clinical Skills Practice Teaching Center, Shengjing Hospital of China Medical University, Shenyang, China

**Keywords:** Congenital adrenal hypoplasia, Hypogonadotropic hypogonadism, DAX1 gene

## Abstract

**Background:**

Most patients with congenital adrenal hypoplasia (AHC) develop symptoms during infantile and juvenile periods, with varying clinical manifestations. AHC is a disease that is easily misdiagnosed as Addison’s disease or congenital adrenal hyperplasia (CAH). There was also a significant time difference between the age at which patients developed symptoms and the age at which they were diagnosed with AHC. Most patients showed early symptoms during infantile and juvenile periods, but were diagnosed with AHC many years later.

**Case presentation:**

We are currently reporting a male patient who developed systemic pigmentation at age 2 and was initially diagnosed with Addison’s disease. At 22 years of age, he experienced a slipped capital femoral epiphysis (SCFE), a disease mostly seen in adolescents aged 8–15 years, an important cause of which is endocrine disorder. Testes evaluated using color Doppler Ultrasonography suggested microcalcifications. Further genetic testing and auxiliary examinations revealed that the patient had hypogonadotropic hypogonadism (HH) and DAX-1 gene disorders, at which time he was diagnosed with AHC complicated by HH. He was given hormone replacement therapy, followed by regular outpatient review to adjust the medication.

**Conclusions:**

The typical early symptoms of AHC are hyperpigmentation and ion disturbance during infantile and juvenile periods, while few patients with AHC develop puberty disorders as early symptoms. AHC is prone to being misdiagnosed as Addison’s disease, and then gradually develops the symptoms of HH in adolescence. The definitive diagnosis of AHC ultimately is based on the patient’s clinical presentation, laboratory results and genetic testing results.

## Background

Congenital adrenal hypoplasia (AHC), also known as X-linked AHC, is a rare familial adrenocortical hypoplasia that was first reported in 1948 [1, 2]. The incidence reported abroad is approximately l:12500 [3], but to date there have been no reports of its incidence in China. The first Chinese patient with AHC was reported in 2007 [4]. The pathological feature of AHC is the absence of permanent cortical areas in the adrenal cortex, which are replaced by large vacuolated cells. AHC can be associated with hypogonadotropic hypogonadism (HH) [5, 6],which is associated with low gonadotropins and testosterone levels. In terms of clinical manifestations, AHC is primarily characterized by adrenocortical hormone deficiency and hypogonadotropic hypogonadism (HH), and its molecular pathology typically reveals DAX-1 mutations. The DAX-1 gene is a dose-sensitive sex reversal, adrenal hypoplasia critical region on chromosome X-gene 1 [7], also known as NROB1 (nuclear receptor subfamily 0, group B, member 1). NROB1 is widely expressed in adrenal cortical cells, leydig cells, and sertoli cells in testes, spermatogenic cells, pituitary gonadotrophic cells, and ventral medial nuclear cells of the thalamus [8]. NROB1 is associated with cell proliferation and differentiation and may play a role in the synthesis of steroid hormones. Therefore, mutations may lead to adrenal cortical development disorders, steroid hormone synthesis deficiency, development disorders of hypothalamic GnRH cells, luteinizing hormone (LH) and follicle stimulating hormone (FSH) cells in the pituitary gland, and testicular dysplasia [9, 10].

In recent years, continuous advances in diagnosis and treatment of AHC have contributed to an increasing number of cases diagnosed, but lack of knowledge regarding AHC may lead to misdiagnosis as congenital adrenal hyperplasia (CAH), aldosterone deficiency, or Addison’s disease, which may result in patients experiencing delays in treatment, as well as improperly medicated [11]. We are currently reporting on a male patient who developed systemic pigmentation at age 2 and was initially diagnosed with Addison’s disease. At the age of 22, he experienced a slipped capital femoral epiphysis (SCFE). The incidence of SCFE was 1 ~ 24.6/100,000, mostly occurring in children aged 8–15 years [12, 13]. Among its many etiologies, endocrine disorder is an important cause, including changes in the levels of sex hormones, growth hormones, and other hormones [14]. Further genetic testing and auxiliary examinations revealed that the patient had HH and DAX-1 gene disorders, at which time he was diagnosed with AHC complicated by HH. As part of the current investigation related cases have been reviewed and their clinical manifestations, laboratory, and genetic monitoring results have been summarized, with the aim of improving clinicians’ understanding of AHC, avoiding misdiagnosis, and providing assistance to family members of patients during prenatal consultations.

## Case report

A male patient aged 22 years was admitted to our endocrinology department in August 2019 due to “20 years of systemic pigmentation”. Briefly, at the age of 2, the patient developed purple lips and gums without inducement, accompanied by a lack of strength and a poor appetite. Upon examination (details are unknown), he was treated for “Addison’s disease” in a pediatric outpatient hospital clinic and received irregular treatment with hydrocortisone by oral administration. At 12 years of age, the patient reported a lack of strength and visited Shanghai Ruijin Hospital for treatment. Based on related auxiliary examinations (specific test results are unknown), he was diagnosed with “Addison’s disease”. At the pubertal stage, the patient received an experimental GnRH pump treatment during his stay in Ruijin Hospital, and chorionic gonadotropin was intramuscularly injected after discharge (75 units twice per week).

The patient discontinued treatment 1 year later. In February 2019, the patient suffered from SCFE and underwent an open reduction and screw fixation. The patient’s height during infancy was slower than that of children of the same age but increased from 1.5 m to 1.78 m between 2014 and 2019. The patient reported that his growth had not stopped at present, and the genitals of the patient were smaller than that of children of the same age, presenting as a small scrotum and micropenis. The patient was therefore admitted to our endocrinology department in August 2019 for 1) adrenocortical hypofunction; 2) gonadal dysgenesis, the causes of which are yet to be investigated; and 3) postoperative of SCFE. The patient has occasionally experienced a lack of strength, inappetence, nausea and vomiting. Additional patient history includes full-term normal delivery and healthy parents, and denial of a similar family history.

Physical examination upon admission: temperature 36.0 °C; pulse 70 beats/min; blood pressure 106/78 mmHg; respiratory rate 18 breaths/min, height 1.78 m, weight 63 kg, systematic pigmentation, darkened lips and gums, obvious pigmentation at the limbs and joints, no development in Adam’s apple, beard and breasts, no armpit and pubic hair, infantile genitalia (TANNER 1) and perineum (TANNER1–2), penis length 4 cm, testicle volume 3 ml (left) and 4 ml (right), no involvement in the thyroid gland, no large heart border, no pathological murmur in each valve, no involvement in the abdomen, liver, and spleen, absence of tenderness and rebound tenderness, and no swollen lower extremities.

**Fig. 1:**
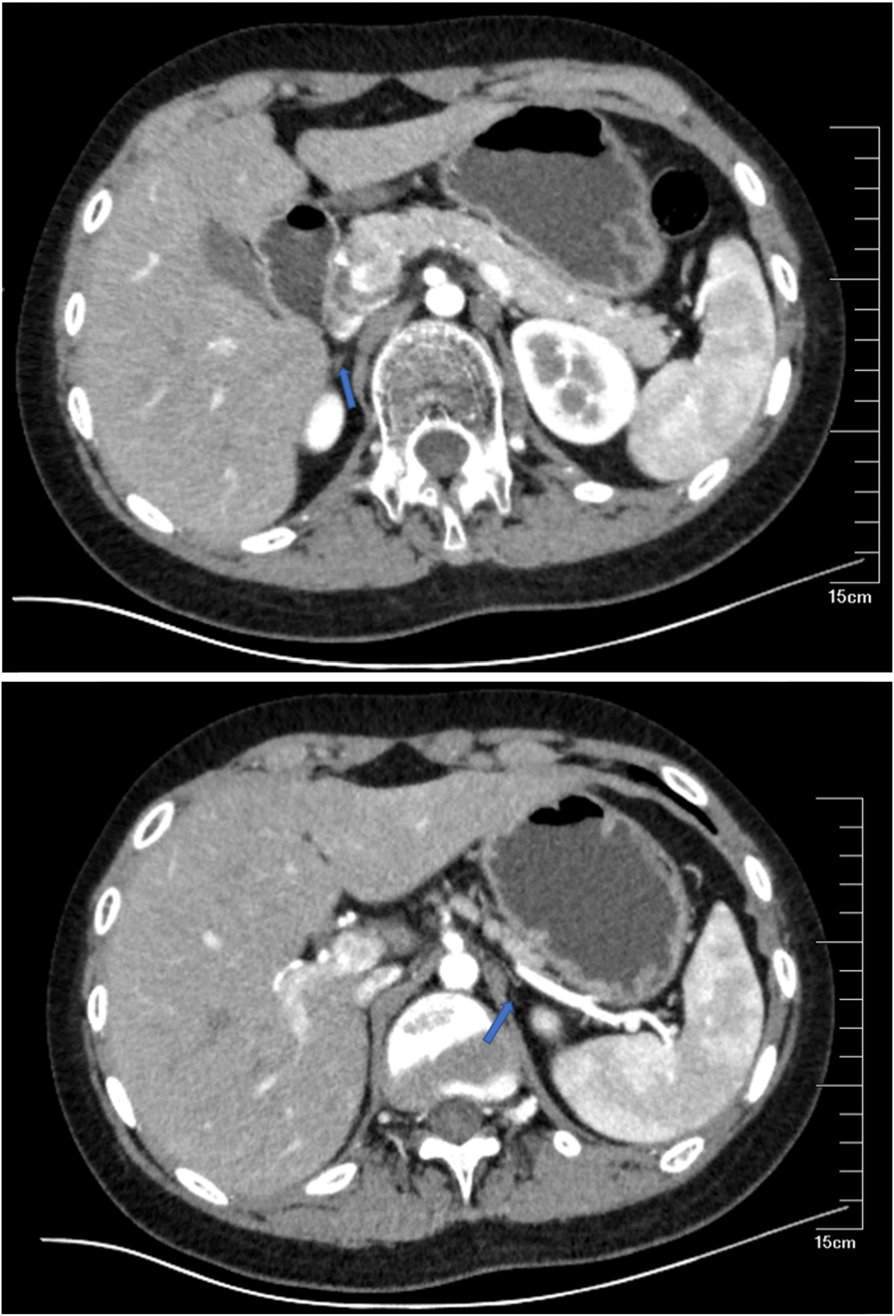
The results of bilateral adrenal enhanced CT

Laboratory examination(performed during the administration of hydrocortisone): PTH: 37.53 pg/mL(15.0–68.3 pg/mL); thyroid functions: TSH 1.16 (0.30–4.8 miu/mL), FT3 3.98 (2.63–5.71 pmol/L), FT4 12.28 (9.01–19.05 pmol/L); blood routine: white blood cell count 8.32 × 109/L, red blood cell count 4.7 × 1012/L, hemoglobin 128 g/L, platelets 180 × 109/L; 25-OHVitD:10.8 ng/mL; blood glucose (fasting):8.0 mU/L,HbA1c5.2;urine microalbumin/urinary creatinine 2.5 mg/g. 24-h urinary free cortisol determination: 24-h urine free cortisol concentration 15.1 ng/mL(2.3–30.0 ng/mL), 24-h urine volume 2000.0 ml, 24-h urinary free cortisol content 30.2 μg/24 h(≥18 years of age,3.5–45.0 μg/24 h); sex hormones: E2 < 20 pg/mL, FSH 1.22 (1.27–19.26miu/mL), LH < 0.2 (1.24–8.62miu/mL), PRL 5.68 (2.64–13.13 ng/mL), progesterone(P) < 0.08 (0.1–0.84 ng/mL), testosterone(T) 1.45 (1.75–7.81 ng/mL), GH 0.063 (0.004–1.406 ng/ mL), IGF-1152.8 (60–350 ng/mL), IGFBP-3 6.87 (3.4–7.8 μg/mL), sex hormone-binding globulin(SHBG)38.0 (13.3–89.5 nmol/L), DHEA-S 166.8 (85–690 μg/dL), free androgen index 13.24 (24.3–110.2%), F-T 2.02 (15–50 pg/mL), serum androstenedione < 0.30 (0.6–3.1 ng/mL), 17a-hydroxyprogesterone(17α-OHP) 0.14 (0.31–2.13 ng/mL). Renin-angiotensin-aldosterone experiment: plasma renin activity 25.917 (0.10–6.56 (ng/mL)/hour), aldosterone 64.45 (70–300 pg/mL). Enhanced CT indicated bilateral adrenal hypoplasia (Fig. 1). Testes evaluated using color Doppler Ultrasonography suggested microcalcifications. The epiphyseal line was unclosed, with a bone age 12.5 years (10 years later than the actual age), bone density T-score − 4.5 and Z-score − 4.5. The results of the cortisol rhythm test (Table 1) showed a significant increase in ACTH and a decrease in cortisol, consistent with adrenocortical hypofunction. The patient underwent a gonadorelin stimulation test (Gonadorelin injection 0.1 mg + saline 10 ml intravenously) and a gonadorelin extension test (Gonadorelin injection 0.1 mg + water injection 1 ml subcutaneously for 4 days, Gonadorelin injection 0.1 mg + saline 10 ml intravenously on the fifth day). The results (Table 2) suggested that the hypothalamic-pituitary-gonad axis (HPG axis) was not activated. Human chorionic gonadotropin (HCG) stimulation test (Table 3) suggested that testicular stromal cells were present, but the level of testosterone was low. After obtaining the consent of the patient and his family, further genetic testing was performed on the patient and his mother. The patient’s DAX-1 gene test result indicated that p.C202Sfs*62 was pathogenic, while his mother’s indicated that she was a carrier of a heterozygous p.C202Sfs*62.

**Table 1:** Results of the cortisol rhythm experiment

time	0:00	8:00	16:00
corticotropin (ACTH), pg/mL	34.9	555.1	1792.0
cortisol, ug/dL	4.0	1.2	0.9

**Table 2:** Results of gonadorelin stimulation test and gonadorelin extension test

	Gn-RH stimulation test		Gn-RH extended test	
Time(min)	FSH IU/L	LH IU/L	FSH IU/L	LH IU/L
0	1.79	0.40	5.12	0.05
30	2.02	0.74	9.39	2.27
60	2.19	1.01	13.96	3.17
90	2.88	1.40	11.29	2.73
120	3.86	1.34	11.86	2.49

**Table 3:** Results of chorionic gonadotropin (HCG) stimulation experiment

	HCG stimulation test
Time(h)	T(ng/ml)
0	0.3
24	0.5
48	0.7
72	1.0

In combination with the patient’s past history, clinical manifestations, auxiliary examinations, and results of genetic testing, the patient was diagnosed with 1) AHC complicated by HH; 2) vitamin D deficiency; 3) osteoporosis; and 4) postoperative of SCFE.

The patient was treated with: 1. Before admission, the patient was given hydrocortisone 40 mg once daily, oral administration. The results of biochemical examination after admission indicated that the level of ACTH was higher, and the level of COR and aldosterone were lower. At the same time, the patient complained of obvious eyelid edema in the morning after taking the present dose of hydrocortisone, accompanied by fatigue, loss of appetite, nausea and vomiting. Dexamethasone is a long-acting corticosteroid. On the one hand, its effect on water and salt metabolism is almost zero, and on the other hand, it has the strongest inhibitory effect on the hypothalamic–pituitary–adrenal axis (HPA). Therefore, combined with the fact that the patient has symptoms such as eyelid edema in early morning and ACTH is significantly higher than the normal, we decided to give the patient dexamethasone orally for a short time and reduce the dose of hydrocortisone. Hormone replacement therapy including:hydrocortisone,0.5tablet(10 mg),once daily in the morning, oral administration; dexamethasone, 1 tablet (0.75 mg), once daily in the evening, oral administration; and fludrocortisone, 0.5 tablet (0.05 mg), once daily, oral administration; however, the patient was unable to purchase fludrocortisone, administered hydrocortisone,1 tablet(20 mg,10 mg in the morning, 5 mg in the afternoon, 5 mg in the evening),oral administration and 1 tablet (0.75 mg) of dexamethasone orally once daily in the evening. After 3 days of treatment, the patient reported that edema symptoms in the morning were alleviated, and ACTH was significantly decreased compared with the previous. Therefore, dexamethasone was discontinued .2. Considering the diagnosis of HH, gonadotropin 2000 iu + 2 ml sodium chloride was administered to the patient twice a week via intramuscular injection. 3.Calcium and vitamin D supplements: Caltrate D2 tablets (1200 mg), once daily, oral administration; 6 vitamin D capsules (2400 iu), once daily, oral administration.

After 3 months of treatment, the patient was re-examined in November 2019. The patient is in good condition, with blood pressure 126/74 mmHg, reduced skin pigmentation, occasional fatigue, and no discomfort such as nausea and vomiting. Laboratory examination: F-T 1.07(15-50 pg/mL), serum androstenedione< 0.30(0.6–3.1 ng/mL),serum potassium (K+)4.83(3.5–5.5 mmol/L),serum sodium(Na+)134(136-145 mmol/L),Renin-angiotensin-aldosterone experiment: plasma renin activity 7.89 (0.10–6.56 (ng/mL)/hour), aldosterone 64.450 (70–300 pg/mL). The patient’s LH and FSH levels increased compared to pre-treatment measurements (Table 4). The patient received cortisol rhythm determination: ACTH (8:00) 1914 (7.2–63.3 pg/mL), COR1.41 (6.02–18.4 μg/dL). Because the patient still has fatigue symptoms, and the serum sodium and aldosterone are lower than the normal, it is suggested that the patient should take hydrocortisone (10 mg in the morning, 5 mg in the afternoon and 5 mg in the evening), plus fludrocortisone (0.05 mg) once a day to improve the aldosterone deficiency. At the same time, gonadotropin 2000 iu + 2 ml sodium chloride were continued to be injected intramuscular twice a week.

**Table 4:** Results of sex hormone examination of patients after treatment

sex hormone	the result
E2	< 20 pg/ml
FSH	2.60 IU/L
LH	< 0.2 IU/L
P	< 0.08 ng/ml
T	0.78 ng/ml

The patient was re-examined in April 2020. The patient’s mental state was satisfactory, and the blood pressure was within the normal range. The skin pigmentation was less than before, and there was no discomfort such as fatigue, nausea, vomiting. Blood electrolyte, ACTH, renin and aldosterone were in the normal range, and blood cortisol was slightly lower than the lower limit of normal. Therefore, hydrocortisone (10 mg in the morning, 5 mg in the afternoon, 5 mg in the evening) and fludrocortisone (0.05 mg, once daily) were continued to be given to the patient.

## Discussion and conclusions

### Early symptoms of AHC

Patients with AHC usually develop symptoms during infantile and juvenile periods, typically manifesting as skin pigmentation or various other systemic signs caused by severe plasma disturbances of hyperkalemia and hyponatremia, such as nausea, vomiting, lack of strength, poor appetite, severe dehydration, respiratory distress, and occasionally puberty disorders. We searched PubMed database, Chinese Journal Full-Text Database (CNKI), British Medical Journal Publishing Group. BMJ and other databases by computer to collect AHC literature. Search Words included “congenital adrenal hypoplasia; hypogonadotropic hypogonadism; DAX1 genes “. All references to included reports and relevant reviews were screened manually for additional potential eligible cases. Finally, we reviewed 21 pieces of literature [15–35] which included a total of 26 cases of AHC and is stratified by the patients’ age of onset and early symptoms, as well as the age at which they were diagnosed with AHC (Table 5). Of the 26 cases collected, 8 had hyperpigmentation complicated by ion disturbances [15, 16, 19, 20, 25, 27, 29, 31], 4 had hyperpigmentation [17, 18, 22, 32], 10 had ion disturbances [24–26, 28, 29, 31, 33, 34], 3 had puberty disorders [21, 23, 35]. Most AHC patients developed early symptoms of hyperpigmentation, ion disturbances, and few developed puberty disorders as early symptoms. There was also a significant time difference between the age when patients developed symptoms and the age at which they were diagnosed with AHC. Most patients showed early symptoms during infantile and juvenile periods, but were diagnosed with AHC many years later. Specific examples include a patient who developed hyperpigmentation and ion disturbances at age 9 but was diagnosed with AHC at age 24 [19], and a patient who developed hyperpigmentation and ion disturbances at age 4, but was diagnosed at age 17 [31]. Only a small number of patients with a family history [28, 31] received timely genetic testing and were diagnosed when the symptoms first occurred. Special attention should therefore be paid to the likelihood of AHC and genetic testing should be carried out in a timely manner when a patient has symptoms such as hyperpigmentation, severe electrolyte disturbances, puberty disorders, and so on during infantile and juvenile periods. We also found that most of the patients showed adrenal insufficiency (AI)-related manifestations during infantile and juvenile periods, whereas 2 patients reported by Nikolaos et al. [29] didn’t develop symptoms including hyperpigmentation and electrolyte disturbances characterized by low levels of sodium and high levels of potassium until adulthood and were diagnosed with AI. In the follow-up examinations, both patients were found to have androgen deficiency syndromes and were misdiagnosed with HH. Further genetic testing led to a definitive diagnosis of AHC. Therefore, the likelihood of AHC should be considered in patients with symptoms related to adrenal insufficiency in adulthood.

**Table 5:** Classification of the different first symptoms

Author, year	first symptom and time	Initial diagnosis	treatment
Huizhen W,2018 [15]	8 months; Hyperpigmentation, Ion disorder	CAH	Hydrocortisone, Fludrocortisone
Haiyan W,2019 [16]	23 days; Hyperpigmentation, Ion disorder	AHC	Hydrocortisone
Yongpan O,2018 [17]	After birth; Hyperpigmentation	No mention (family history)	Hydrocortisone, Fludrocortisone
Xiaojing L,2016 [18]	1 months; Hyperpigmentation	CAH	Hydrocortisone, Fludrocortisone
Huabing Z,2011 [19]	9 years; Hyperpigmentation, Ion disorder	PAI	Hydrocortisone
Chaohui H,2009 [20]	9 years; Hyperpigmentation, Ion disorder	Addison	Prednisone
Yun L,2011 [21]	13 years; Gonadal dysgenesis	Addison	Prednisone
Lihua Z,2012 [22]	2 years; Hyperpigmentation	Addison	Hydrocortisone
Danping W,2011 [23]	13 years; Gonadal dysgenesis	Addison	Hydrocortisone
Sourabh Verma,2019 [24]	After birth; Secondary epilepsy, Ion disorder	ACH	Hydrocortisone
Bernardo Dias Pereira 2015 [25]	18 days; Hyperpigmentation, Ion disorder	CAH	Glucocorticoid
	14 days,Ion disorder	CAH	Hydrocortisone
C. Frapsauce,2011 [26]	3 weeks;Ion disorder	PAI	Hydrocortisone, Fludrocortisone
Karine Gerster,2017 [27]	2 years and 5 months; Hyperpigmentation, Ion disorder	PAI	Hydrocortisone, Fludrocortisone
Anastasios Serbis,2018 [28]	8 months;Ion disorder	ACH(family history)	Hydrocortisone, Fludrocortisone
Nikolaos Kyriakakis,2017 [29]	19 years; Ion disorder	AI	Hydrocortisone, Fludrocortisone
	30 years; Hyperpigmentation, Ion disorder	AI	Hydrocortisone, Fludrocortisone
Johari Mohd Ali,2014 [30]	11 years and 4 months;Convulsions, High fever,	No mention	Hydrocortisone, Fludrocortisone
Erdem Durmaz,2013 [31]	4 years; Hyperpigmentation, Ion disorder	PAI	Hydrocortisone, Fludrocortisone
	6 years; Ion disorder	ACH (family history)	Hydrocortisone, Fludrocortisone
	9 days; Ion disorder	ACH (family history)	Hydrocortisone, Fludrocortisone
Siyue Liu,2019 [32]	9 years; Hyperpigmentation	PAI	Hydrocortisone
Rita Bertalan,2019 [33]	4 years; Ion disorder	PAI	Glucocorticoid, Testosterone
Tijen Karsli,2016 [34]	6 days; Ion disorder	ACH	Glucocorticoid
Minlian D,2015 [35]	after birth; Ion disorder	ACH	Glucocorticoid
	4 months; Precocious puberty	Adrenal dysplasia, CPP	Hydrocortisone, Fludrocortisone

1.*AHC* congenital adrenal hypoplasia 2.*PAI* primary adrenal insufficiency 3.*AI* adrenal insufficiency 4. *Addison* primary chronic adrenocortical hypofunction 5. *CAH* congenital adrenal hyperplasia 6.*CPP* central precocious puberty

### Diverse clinical manifestations of AHC

AHC typically manifests as primary AI, HH, and impaired fertility [36], and AHC patients tend to suffer of adrenocortical hypofunction during infantile and juvenile periods accompanied by unchanged or decreased bodyweight, refusal to eat, vomiting, diarrhea, dehydration, drowsiness, and skin pigmentation. Blood test results typically show severe low levels of sodium, high levels of potassium, and acidosis [18, 37–40]. Moreover, with age, patients with AHC can show puberty disorders. Some studies have reported that patients with AHC often have symptoms of hypogonadotropic hypogonadism, which is manifested as delayed puberty [41],and a small part of AHC patients can show symptoms of precocious puberty [42–45]. In addition, a small number of AHC patients may also present with other clinical manifestations that are of diagnostic significance, such as: SCFE and Testicular microlithiasis (TM).

### Puberty disorders

#### Delayed puberty

AHC can be associated with hypogonadotropic hypogonadism [5, 6]. Patients with AHC typically develop AI during infantile and juvenile periods and HH during adolescence. The patient reported in the current study developed symptoms of adrenal insufficiency, such as purplish lips and gums at age 2 years. Results from the cortisol rhythm test also suggested a significant increase in ACTH and a decrease in cortisol. With an increased age, the patient was found to have inconsistent secondary sex characteristics, gonad volume, sex hormone level, and bone age compared to his actual age and be in a state of growth retardation. An auxiliary examination indicated low levels of testosterone, FSH, and LH, and the Gonadorelin test results indicated that the HPG axis of the patient could not be activated. Thus, both clinical manifestations and laboratory test results suggested that the patient had AI complicated by HH. In other related studies, delayed puberty has also been reported in AHC patients. Clinical symptoms include: small testicles, delayed development, and low levels of androgens and pituitary gonadotropins [21, 23].

#### Precocious puberty

Many studies [17, 31, 35, 42–45] also reported cases of AHC with precocious puberty. Precocious puberty mainly manifests as premature adrenarche (PA), undue growth of the penis, undue increase in bone age, and undue enlargement of the testes [46, 47]. In 2015, a case with congenital AHC caused by mutations in the DAX-1 gene was reported in China [35]. The case was first described as having central precocious puberty (CPP), but was treated only with cortisol instead of precocious puberty therapy [35]. Domenice et al. [43] reported an AHC patient with precocious puberty, whose GnRH excitement was at prepuberty levels, decreased ACTH after corticosteroid replacement therapy, the testosterone levels returned to prepuberty and the testes no longer increased in size. Thus, the patient was considered to have ACTH-dependent precocious puberty. Although the mechanism of DAX-1-induced precocious puberty remains unclear, adequate corticosteroids should be administered as a preferred therapy for the follow up the changes in precocious puberty rather than immediate use of GnRHa, given that the disease is temporary [17].

#### SCFE

This patient suffered from SCFE after a sprain. SCFE occurs mostly in adolescents aged 10–13 years, and an endocrine disorder is considered to be an important etiological agent— both the absolute lack of sex hormones and the relative excess of growth hormones in adolescents make them prone to cartilage-like fragility and therefore SCFE due to shearing forces under weight-bearing conditions. It has also been reported that changes in hormone levels, hypothyroidism, adrenal hypofunction, and decreased pituitary hormone secretion may increase the incidence of SCFE. Therefore, SCFE may also be used as an indicator of AHC.

#### Testicular microlithiasis (TM)

The current patient’s testicular when evaluated using color Doppler ultrasound showed testicular microcalcifications. Despite unclear pathogenesis, TM is associated with various diseases (eg, cryptorchidism, congenital adrenal hyperplasia, varicocele, and testicular malignancy). Anastasios Serbis et al. [28] first identified an X-linked AHC patient with TM. The patient was an 11-year-old boy who suffered an adrenal crisis at 8 months old and was subsequently diagnosed with AHC, and subsequently developed TM during follow-up. One study [48] reported the relationship between TM and infertility and drew the conclusion that TM may cause infertility. Therefore, the possibility of TM should be considered during the follow-up of AHC patients.

### A high likelihood of the misdiagnosis of AHC

AHC typically manifests as adrenocortical hypofunction in infancy and early childhood and hypogonadism in adolescence. Most patients are not diagnosed with AHC at the time of their initial symptoms and are misdiagnosed with CAH, aldosterone deficiency, and Addison’s disease (Table 5). One case of a 21-day-old baby boy who was admitted to hospital due to feeding difficulties and poor growth has been reported [49]. Physical examination showed skin pigmentation and normal external genitalia, and laboratory tests showed low levels of sodium and high levels of potassium, hypoglycemia, decreased cortisol levels, slightly increased 17-OHP levels, and significantly increased 11-deoxycortisol levels. These results have led to an early diagnosis of CAH. However, the results of the genetic testing suggested missense mutations in the DAX-1 gene, and therefore the patient was later diagnosed with AHC. In 2016, a 12-year-old boy with gonadal dysplasia was reported [18]. The patient was diagnosed with Addison’s disease on the basis of8-year-long repetitively relapse salt-wasting syndrome. The diagnosis of AHC was not made until the patient was 12 years of age when he visited a hospital due to gonad dysplasia. Two misdiagnosed cases (cases 1 and 6) were also included in the 2019 report, which included seven case-child patients. Case 1 was first diagnosed with CAH, but was later found to have significantly increased levels of ACTH, decreased levels of 17a-hydroxyprogesterone and testosterone, as well as retarded growth and bone age. After genetic testing, the patient was diagnosed with AHC [11]. Additional cases that have been misdiagnosed have also been reported [50, 51]. As such, it is of great importance for clinicians to correctly identify AHC, CAH, and Addison’s disease. CAH is an autosomal recessive disorder that affects both males and females, and 21-hydroxylase deficiency is a commonly seen disease type. In addition to salt loss, female patients may have varying degrees of virilization, and male patients are prone to peripheral precocious puberty with homosexual manifestations, elevated levels of 17a- hydroxyprogesterone, testosterone, and androstenedione, and obviously bilateral adrenal hyperplasia upon imaging examination. Unlike CAH, AHC is an X-linked recessive disorder that only affects males, typically manifesting as sexual dysplasia, normal phenotype, normal or decreased level of 17a- hydroxyprogesterone, decreased levels of testosterone and androstenedione, and identifiable atrophy of the adrenal glands. Secondary adrenal insufficiency is generally associated with various primary diseases, and patients with the disease have no family history and can be diagnosed with genetic testing.

### Diagnosis and treatment of AHC

The clinical manifestations of AHC are very diverse. Patients who have developed skin pigmentation, severe hyperkalemia, hyponatremia, and significantly elevated serum ACTH levels should be considered the possibility of AHC. The assessment for AHC should be combined with laboratory tests (for cortisol, 17α-hydroxyprogesterone, testosterone, progesterone, etc.) and genetic testing to avoid missed diagnosis and misdiagnosis, of which genetic testing is considered to be a reliable means to support a diagnosis of AHC. In order to facilitate a timely diagnosis and treatment initiation, women and men of reproductive age whose family member has been diagnosed with AHC should receive genetic testing for DAX1/NROB1. Early diagnosis and treatment should be performed for patients with AHC to improve their quality of life and to prevent adrenal crisis. In addition, Johari et al. [30] reported that the level of free estriol (E3) in the serum of pregnant women during the third trimester was associated with fetal AI. Jasmina [52] concluded that the detection of E3 concentrations in the serum of pregnant women during pregnancy contributes to the diagnosis of AHC, suggesting that E3 is derived from dehydroepiandrosterone (DHEA) synthesized in the fetal adrenal glands. In the case of AI, the excretion of fetal DHEA is impaired, resulting in a decrease in the concentration of E3 in pregnant women, which is apparent in women who have delivered a child with AHC. Therapies for patients with AHC include glucocorticoid replacement, mineralocorticoid replacement, and promotion of development and fertility. As for the glucocorticoids used for AHC treatment, short-acting preparations (cortisone or hydrocortisone) are preferred and they should be administered according to the cortisol secretion rhythm. Sufficient intake of mineralocorticoids should be ensured and should last for a lifetime. In terms of promoting development and fertility, given that AHC patients commonly suffer from infertility due to poor gonadotropin response and limited spermatogenic ability in their testes, the goal of treatment is to promote the development of male secondary sexual characteristics and epiphyseal closure, to relieve osteoporosis, and preventing cardiovascular diseases. Therapies including oral administration/injection of testosterone and skin patches are generally recommended and the initial age of treatment is preferably 16–18 years of age [53]. With respect to developmental height retardation in AHC patients, some studies have suggested that appropriate glucocorticoids (GC) and mineralocorticoid (MC) replacements are clearly important to maintain normal growth velocity [54].

In summary, the clinical manifestations of AHC are diverse and vary in severity. Due to the deficiency of both glucocorticoids and mineralocorticoids secreted by the adrenal glands, most patients with AHC develop severe dehydration and electrolyte disturbances during infantile and juvenile periods. AHC is prone to misdiagnosis as CAH, aldosterone deficiency, and Addison’s disease, and may results into death if hormone replacement therapy is not initiated in a timely manner. As patients age most of them develop varying degrees of HH at adolescence. Therefore, boys who have unexplained primary adrenocortical dysfunction complicated by HH, should receive DAX-1 mutation screening in order to make a diagnosis of AHC. Collectively, these factors will further improve understanding of the clinical manifestations and molecular pathology of AHC in child patients.

## Data Availability

All authors declare that data and any supporting material regarding this manuscript is available and it can be requested at any time.

## Abbreviations

AHC: Congenital adrenal hypoplasia
HH: Hypogonadotropic hypogonadism
CAH: Congenital adrenal hyperplasia
SCFE: Slipped capital femoral epiphysis
NROB1: Nuclear receptor subfamily 0, group B, member 1
LH: Luteinizing hormone
FSH: Follicle stimulating hormone
TSH: Thyroid stimulating hormone
PTH: Parathyroid hormone
FT: Free triiodothyronine
HbA1c: Glycosylated haemoglobin
PRL: Prolactin
T: Testosterone
P: Progesterone
GH: Growth hormone
IGF-1: Insulin-like growth factor 1
IGFBP-3: Insulin-like growth factor binding protein 3
SHBG: Sex hormone-binding globulin
F-T: Free testosterone
17α-OHP: 17a-hydroxyprogesterone
CT: Computed tomography
ACTH: Adrenocorticotropic hormone
HPG: Hypothalamic-pituitary-gonad axis
HCG: Human chorionic gonadotropin
COR: Cortisol
HPA: Hypothalamic–pituitary–adrenal axis
AI: Adrenal insufficiency
TM: Testicular microlithiasis
CPP: Central precocious puberty
E3: Free estriol
GC: Glucocorticoids
MC: Mineralocorticoid
PAI: Primary adrenal insufficiency

## References

[1] Sikl H (1948). Addison’s disease due to congenital adrenal hypoplasia of the adrenals in an infant aged 33 days. J Pathol Bactertiol.

[2] Zanaria E, Muscatelli F, Bardoni B (1994). An unusual member of the nuclear hormone receptor superfamily responsible for X-linked adrenal hypoplasia congenita[J]. Nature.

[3] Jadhav U, Harris RM, Jameson JL (2011). Hypogonadotropic hypogonadism in subjects with DAXJ mutations. Mol Cell Endocrinol.

[4] Xiao Y, Yang J, Zhang HJ (2007). Identification of a novel missense mutation of the DAX-J gene in a Chinese pedigree with X—linked adrenal hypoplasia congenital [J]. Chin J Pediatr.

[5] Muscatelli F, Strom TM, Walker AP, Zanaria E, Récan D, Meindl A, Bardoni B, Guioli S, Zehetner G, Rabl W, Peter Schwarz H, Kaplan JC, Camerino G, Meitinger T, Monaco AP (1994). Mutations in the DAX-1 gene give rise to both X-linked adrenal hypoplasia congenita and hypogonadotropic hypogonadism. Nature..

[6] Landau Z, Hanukoglu A, Sack J, Goldstein N, Weintrob N, Eliakim A, Gillis D, Sagi M, Shomrat R, Kosinovsky EB, Anikster Y (2010). Clinical and genetic heterogeneity of congenital adrenal hypoplasia due to NR0B1 gene mutations. Clin Endocrinol.

[7] Lihua Y, Yuci L, Xunliang W (2014). Et a1. Study on the dose-sensitive interaction between androgen receptor and DAX-1[J]. Chin J Andrology.

[8] Landau Z, Hanukoglu A, Sack J, Goldstein N, Weintrob N, Eliakim A, Gillis D, Sagi M, Shomrat R, Kosinovsky EB, Anikster Y (2010). Clinical and genetic heterogeneity of congenital adrenal hypoplasia due to NR0B1 gene mutations. Clin Endocrinol.

[9] Ehrlund A, Treuter E (2012). Ligand-independent actions of the orphan receptors/corepressors DAX-1 and SHP in metabolism, reproduction and disease[J]. J Steroid Biochemistry Mol Biol.

[10] Zheng JJ, Wu XY, Nie M (2016). Dysfunction of hypothalamic-pituitary-testicular axis in patients with adrenal hypoplasia congenita due to DAX—l gene mutation[J]. Natl Med J China.

[11] Qiong C, Haiyan W (2019). Clinical and molecular genetic characterizations of 7 children with X—linked adrenal hypoplasia congenita[J]. Chin J Appl Clin Pediatr.

[12] Loder RT, Skopelja EN (2011). The epidemiology and demographics of slipped capital femoral epiphysis[J]. ISRN Orthop.

[13] Larson AN, Yu EM, Melton LJ (2010). Incidence of slipped capital femoral epiphysis: a population-based study[J]. J PediatrOrthop B.

[14] Hao L, Ziming Z (2018). Recent advances in diagnosing and treating slipped capital femoral epiphysis[J]. Chin J Pediatr Surg.

[15] Huizhen W, Haiyan W, Wenjing W (2018). A case of congenital adrenal dysplasia was diagnosed[J]. Shandong Med.

[16] Huizhen W, Haiyan W (2019). A case of congenital adrenal dysplasia caused by a new mutation of NROB1 gene and literature review[J]. Chin J Practical Pediatr.

[17] Yongpan O. Clinical analysis of 4 cases of congenital adrenal dysplasia with precocious puberty caused by dax-1 mutation[J].Chongqing medical university.

[18] Xiaojiang L, Haihua Y (2016). Et a1.The NROB1 gene missense mutation causes congenital adrenal dysplasia: a case report[J].Journal of. Clin Pediatr.

[19] Huabing Z, Min N (2011). Clinical study and inheritance of protopathic superior renal gland development with hypotropic actin-induced glandular insufficiency[J]. J Reprod Med.

[20] Chaohui H, Chaohui L (2009). X-linked congenital adrenal dysplasia: a case report[J]. Medical Journal of Chinese People's Liberation Army.

[21] Yun L, Heng S (2011). Dax-1 gene mutation caused congenital adrenal dysplasia: a case report Medical[J]. China Medical Engineering.

[22] Lihua Z, Fan P, et a1.Dax-1 gene mutation caused congenital adrenal dysplasia :A case report and literature review[J].Clinical Focus,2012,27(22):1997–1998.

[23] Danping W, Cunren C, et a1. One case of late: onset adrenal hypoplasia congenita caused by a novel mutation of DAX-1 gene[J].Chin J Endocrinal Metab,2011,27(1):47–49.

[24] Sourabh Verma, Sheryl Purrier, Emily Breidbart, et a1. Hyponatremic Seizures and Adrenal Hypoplasia Congenita in a Neonate with Congenital Diaphragmatic Hernia[J].Case Reports in Pediatrics, Volume 2019, Article ID 4178251, 4 pages. 10.1155/2019/4178251, 2019, 4. 10.1155/2019/4178251 PMC6556792 31263616

[25] Bernardo Dias Pereira, et a1.Iris Pereira 2X-linked adrenal hypoplasia congenita: clinical and follow-up findings of two kindreds, one with a novel NR0B1 mutation[J]. Arch Endocrinol Metab. 2015;59(2):181–185. 10.1590/2359-3997000000032 25993682

[26] C. Frapsauce, C. Ravel, M. Legendre, et a1.Birth after TESE–ICSI in a man with hypogonadotropic hypogonadism and congenital adrenal hypoplasia linked to a DAX-1 (NR0B1) mutation[J].Human Reproduction, 2011,26 (3):724–728. 10.1093/humrep/deq372 PMC3037794 21227944

[27] Karine Gerster, Claudia Katschnig, Sascha Wyss, et a1.A novel DAX-1 (NR0B1) mutation in a boy with X-linked adrenal hypoplasia congenita[J].J Pediatr Endocrinol Metab,2017. 10.1515/jpem-2017-0261 29087957

[28] Anastasios Serbis, Vassiliki Regina Tsinopoulou,et a1.Testicular microlithiasis in a boy with X-linked adrenal hypoplasia congenita[J].Ann Pediatr Endocrinol Metab 2018,23:162–165. 10.6065/apem.2018.23.3.162 PMC6177664 30286574

[29] Nikolaos Kyriakakis, Tolulope Shonibare, et a1.Late-onset X-linked adrenal hypoplasia (DAX-1, NR0B1): two new adult-onset cases from a single center[J]. Pituitary,2017: DOI 10.1007/s11102-017-0822-x. 10.1007/s11102-017-0822-x PMC5606946 28741070

[30] Johari Mohd Ali, Muhammad Yazid Jalaludin, et a1. Late onset X-linked adrenal hypoplasia congenital with hypogonadotropic hypgonadism due to a novel 4-bp deletion in exon 2 of NR0B1[J]. J Pediatr Endocr Met 2014; 27(11–12): 1189–1192. 10.1515/jpem-2014-0161 25003377

[31] Erdem Durmaz, Doga Turkkahraman, et a1. A novel DAX-1 mutation presented with precocious puberty and hypogonadotropic hypogonadism in different members of a large pedigree[J]. J Pediatr Endocr Met 2013;26(5–6):551–555. 10.1515/jpem-2012-0086 23585174

[32] Siyue Liu, Libin Yan, Xinrong Zhou, et a1. Delayed-onset adrenal hypoplasia congenita and hypogonadotropic hypogonadism caused by a novel mutation in DAX1[J].Journal of International Medical Research,2019: DOI: 10.1177/0300060519882151, 48, 2, 030006051988215. 10.1177/0300060519882151 PMC7605007 31642359

[33] Bertalan R, Bencsik Z, Mezei P (2019). Et a1. Novel frameshift mutation of the NR0B1(DAX1) in two tall adult brothers[J]. Mol Biol Rep.

[34] Karsli T, Sutter J. et a1, X-linked Adrenal Hypoplasia Congenita Due to NR0B1 (DAX1) Deficiency Presenting as Severe Respiratory Distress in Near Term Infants[J]. Pediatr Neonatology. 2016:1–2. 10.1016/j.pedneo.2015.10.010 27026067

[35] Minlian D, Yanhong L (2015). Children with congenital adrenocortical dysplasia caused by a new mutation in the NROB1 gene have central precocious puberty[J]. Chin J Endocrinology Metab.

[36] Suntharalingham JP, Buonocore F, Duncan AJ (2015). Et a1. DAX-1(NROBl) and steroidogenic factor-1(SF一1, NR5A1)in human disease[J]. Best Pract Res Clin Endocrinol Metab.

[37] Min X, Youming W, et a1.An x-linked congenital adrenal dysplasia caused by a dax-1 gene transcoding process (428delG) [J].Chin J Med Genet, 2009,26(1):11–15.

[38] Jinlei Y, Huijuan Z, Yanxia L (2016). Two cases of congenital adrenal dysplasia with hypogonadotropin hypogonadism[J]. Henan Medical Res.

[39] Bizzarri C, Olivini N, Pedicelli S, Marini R, Giannone G, Cambiaso P, Cappa M (2016). Congenital primary adrenal insufficiency and selective aldosterone defects presenting as salt-wasting in infancy: a single center 10-year experience[J]. Ital J Pediatr.

[40] Yu D (2016). J uan L, Yongnian S, et a1.Etiological analysis and application value of gene diagnosis in 4 cases of infant salt loss syndrome[J].Chinese journal of practical. Pediatrics.

[41] Maohua G, Guijun Q, Xialian L, et a1. Novel DAX-1 mutations in two Chinese families with X-linked congenital adrenal hypoplasia[J].Henan Med Res, 2013,22(6):801–804.

[42] Mantovani G, De Menis E, Borretta G (2006). DAX-1 and X-linked adrenal hypoplasia congenita: clinical and molecular analysis in five patients [J]. Eur J Endocrinol.

[43] Domenice S, Latronico AC, Brito VN, Arnhold IJP, Kok F, Mendonca BB (2001). Adrenocorticotropin-dependent precocious puberty of testicular origin in a boy with X-linked adrenal hypoplasia congenita due to a novel mutation in the DAX1 gene[J]. J Clin Endocrinol Metab.

[44] Amaia Rodríguez Estévez , Gustavo Pérez-Nanclares, et a1.Clinical and molecular characterization of five Spanish kindreds with X-linked adrenal hypoplasia congenita: atypical findings and a novel mutation in NR0B1[J].J Pediatr Endocrinol Metab 2015; 28(9–10): 1129–1137. 10.1515/jpem-2014-0472 26030781

[45] Shima H, Yatsuga S, Nakamura A (2016). Et a1.NR0B1 frameshift mutation in a boy with idiopathic central precocious puberty[J]. Sex Dev.

[46] Nagel SA, Hartmann MF, Riepe FG (2018). Et a1. Gonadotropin—and adrenocorticotropic hormone—independent precocious puberty of gonadal origin in a patient with adrenal hypoplasia congenita due to DAXl gene mutation—a case report and review of the literature:implications for the pathomechanism [J]. Horm Res Paediatr.

[47] Landau Z, Hanukoglu A, Sack J (2010). Clinical and genetic heterogeneity of congenital adrenal hypoplasia due to NROBl gene mutations[J]. Clin Endocrinol.

[48] Aizenstein RI, DiDomenico D, Wilbur AC, O'Neil HK (1998). Testicular microlithiasis: association with male infertility[J]. J Clin Ultrasound.

[49] Flint JL, Jacobson JD. Adrenal hypoplasia congenita presenting as congenital adrenal hyperplasia[J]. Case Rep in Endocrinol. 2013;(6507):393584. 10.1155/2013/393584 PMC3583052 23476826

[50] Wang CL, Fen ZW, Liang L (2014). A de novo mutation of DAX1 in a boy with congenital adrenal hypoplasia without hypogonadotropic hypogonadism[J]. J Pediatr Endocrinol Metab.

[51] Landau Z, Hanukoglu A, Sack J, Goldstein N, Weintrob N, Eliakim A, Gillis D, Sagi M, Shomrat R, Kosinovsky EB, Anikster Y (2010). Clinical and genetic heterogeneity of congenital adrenal hypoplasia due to NROB1 gene mutations[J]. Clin Endocrinol.

[52] Durkovi J, Milenković T, Krone N (2014). Low Estriol Levels in the Maternal Marker Screen as a Predictor of X-Linked Adrenal Hypoplasia Congenita: Case Report[J]. Srp Arh Celok Lek.

[53] Shouyue S, Jun Y (2010). Three cases of congenital adrenocortical hypoplasia with hypogonadotropin gonadal hypoplasia caused by DAXl gene mutation[J]. Nat Med J China.

[54] Minnetti M, Caiulo S, Ferrigno R, Baldini-Ferroli B, Bottaro G, Gianfrilli D, Sbardella E, de Martino MC, Savage MO (2020). Abnormal linear growth in paediatric adrenal diseases: pathogenesis, prevalence and management[J]. Clin Endocrinol.

